# Does Sensory Retraining Improve Subjective Rating of Sensory Impairment after Bilateral Sagittal Split Osteotomy?

**DOI:** 10.1097/GOX.0000000000001769

**Published:** 2018-05-14

**Authors:** Yea-Ling Yang, Ellen Wen-Ching Ko, Yu-Ray Chen, Chiung Shing Huang

**Affiliations:** From the * Graduate Institute of Craniofacial and Dental Science, College of Medicine, Chang Gung University, Taoyuan, Taiwan;; † Department Craniofacial Orthodontics, Craniofacial Research Center, Chang Gung Memorial Hospital, Taipei, Taiwan;; ‡ Department of Plastic and Reconstructive Surgery, Craniofacial Research Center, Chang Gung Memorial Hospital, Linkou, Taiwan.

## Abstract

**Background:**

Sensory retraining could ameliorate neurosensory disturbance (NSD) resulting from the injury of the inferior alveolar nerve after a bilateral sagittal split osteotomy (BSSO). The aim of this study was to measure the effects of sensory retraining on NSD.

**Methods:**

A randomized, prospective, single-blinded clinical trial was conducted on consecutive patients with NSD resulting from a BSSO to correct skeletal class III malocclusion. The outcome measurement of sensory recovery was performed using a patient-centered survey assessed by the total score of questionnaire (TSQ) and visual analogue scale (VAS) at preoperation (T0), 1 week after surgery (T1), each month from 1 to 6 months after surgery (T2–T7), and 1 year after surgery (T8).

**Results:**

In total, 115 consecutive patients were randomized into a control group (56 patients without sensory retraining) or study group (59 patients with sensory retraining). TSQ and VAS values continually decreased from T1 to T8 in both groups. The study group tended to have lower, but not significantly lower, TSQ and VAS values than the control group at each postoperative time point. Women and men responded similarly to sensory retraining at each time point. The oldest patients did not respond well to sensory retraining, compared with the youngest patients, from T1 to T8, and this age effect evaluated by the VAS reached a significant difference at T7 and T8.

**Conclusion:**

NSD, assessed by the TSQ and VAS, continually decreased over time after the BSSO. Sensory retraining could improve NSD, but not significantly.

The bilateral sagittal split osteotomy (BSSO) is the most commonly performed mandibular osteotomy approach. Although it has been modified by Trauner and Obwegeser,^1,2^ its most frequent side effect is a neurosensory disturbance (NSD) of the inferior alveolar nerve (IAN). The incidence of NSD differs depending on the evaluation method. A subjective evaluation method has indicated a higher incidence of NSD than that revealed by the relatively objective sensory test.^3–5^ The percentage of NSD in the lower lip and chin ranges from 29.0% to 100.0% immediately after a BSSO^3,6,7^ and from 11.6% to 61.0% after more than 1 year of follow-up,^3,7,8^ as determined through questionnaires. A subjective evaluation has been the most commonly used method for interpreting neurosensory deficits in previous studies, which is a patient-centered outcome evaluation based on subjective feelings.^9^ The patient-centered survey has been included in a questionnaire and the visual analogue scale (VAS).

Sensory retraining is a rehabilitative approach that helps a patient with a nerve injury to improve the altered sensation or neural impulses reaching his or her conscious level after the stimulation of the altered sensation area.^10^ The purpose of this physiotherapy was to improve the patient’s ability to interpret the altered sensory responses from injured sensory nerves and to improve his or her perception of the function. Studies have investigated mandibular nerve injury through randomized clinical trials; they have found that sensory retraining exercises can lessen objectionable impressions of negatively altered sensations (ie, numbness and loss of sensitivity) at a 2-year follow-up^11–13^ and can alter patient experiences or the way they respond. However, studies on sensory retraining have been conducted by the same medical center, and they have evaluated the same subjects with altered sensations after surgery. The purpose of the current study was to determine whether sensory retraining could significantly reduce NSD after a BSSO. This study tested the hypothesis that sensory retraining could facilitate sensory recovery of NSD resulting from a BSSO. The specific aims are outlined as follows: (1) to measure the long-term effects of sensory retraining on NSD after a BSSO; (2) to assess self-perception of NSD using a questionnaire and the VAS up to 1 year after the BSSO; and (3) to identify factors associated with the effect of sensory retraining.

## PATIENTS AND METHODS

### Study Design

This randomized, prospective, single-blind, clinical trial included consecutive adult patients with skeletal class III malocclusion from the Craniofacial Center at Chang Gung Memorial Hospital (Taoyuan and Taipei branches). The patients’ age ranged from 18 to 45 years, and they underwent a BSSO between May 2012 and February 2014. The inclusion criteria were a skeletal class III jaw relationship and the presence of NSD over the lower lip or chin at postoperative week 1. NSD in this study was defined as patients with a negative sensory outcome according to the touch threshold (ie, von Frey fiber score < 3.84) and pinprick sensory test. The exclusion criteria included a medical condition associated with systemic neuropathy, previous facial surgery or craniofacial trauma, craniofacial anomalies including cleft lip and palate, altered sensation before surgery, and an unwillingness to sign the informed consent form. The study protocol was approved by the Institutional Review Board of Chang Gung Memorial Hospital (100-2302A3), was supported by the National Institute of Health (NMRPD1B0421), and was registered at ClinicalTrials.gov for identification (NCT01586598).

### Study Sample

The patients were enrolled using a 2-arm parallel group design, and the number of patients in each group was equal. Enrollment began in May 2012 and ended in February 2014. The patients underwent a surgery-first approach combined with orthodontic treatment to correct their skeletal disharmony and malocclusion.^14,15^ The surgery was performed by a surgical team comprising 1 team leader and 2 associate surgeons at the Craniofacial Research Center at Chang Gung Memorial Hospital (Taoyuan, Taiwan). All 3 surgeons had been performing orthognathic surgery (OGS) for more than 15 years. The surgical technique (BSSO) was carried out using the modified Obwegeser-Dal Pont technique, as described by Hunsuck.^16^ Three-dimensional cone beam computed tomography images of the pathway of the mandibular nerve were provided during surgery.^17^ The contact area of the mandibular nerve to the buccal cortical plate was indicated in the images. The surgeons may have modified the BSSO dissection to reduce any NSD in the mandibular nerve. Bone fixation was achieved using 2-hole miniplates and 4 monocortical screws (Stryker, Freiburg, Germany) on each side of the retromolar area. Intermaxillary fixation was not used after surgery.

### Randomization

Patients were randomized to 1 of the 2 groups by using block randomization (2A2B) at postoperative week 1. For the allocation of patients, the sequence of randomization was taken from a computer-generated list of random numbers. The patients in both groups were due for a follow-up at postoperative week 1, and then every month from 1 to 6 months, and then 1 year after surgery.

### Programs for Sensory Retraining

For the study group, 3 stages of the sensory retraining exercise were provided during training sessions at 1 week, 1 month, and 3 months after surgery. The exercise started at 1 week after surgery when NSD was detected. The 3 stages of sensory retraining were modified for reeducation, which are commonly used after injuries to the nerves of the hand^18^ (Table 1). The 3 stages of sensory retraining were individually presented by research assistants who were specifically trained and standardized for the sensory evaluation and training used in this research.

**Table 1.**
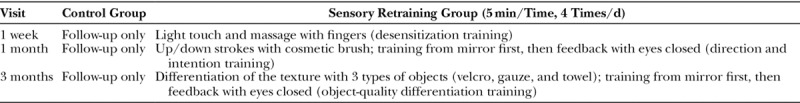
Instructions of the Sensory Retraining Group and Control Group in Each of the Three Retraining Sessions

For the control group, patients were recalled monthly for postoperative orthodontic treatment. Other than regular oral hygiene instruction, no sensory retraining exercise was given to the patients.

### Study Variables

The primary study variable was sensory retraining, divided into 2 groups: the study group with sensory retraining and the control group without sensory retraining. Predictor variables were age (years), sex, and OGS combined with genioplasty or not. Age was further divided into 3 age groups (ie, 17–20, 21–25, and > 25 years) to assess the age effect on sensory retraining. The effect of genioplasty on NSD was also assessed.

### Outcome Measurements

For the patient-centered survey, we evaluated the patients’ subjective sensory disturbance by using a questionnaire and the VAS at preoperation (T0), 1 week after surgery (T1), each month from 1 to 6 months after surgery (T2, T3, T4, T5, T6, and T7), and 1 year after surgery (T8) in both groups.

#### Questionnaire

**Fig. 1.**
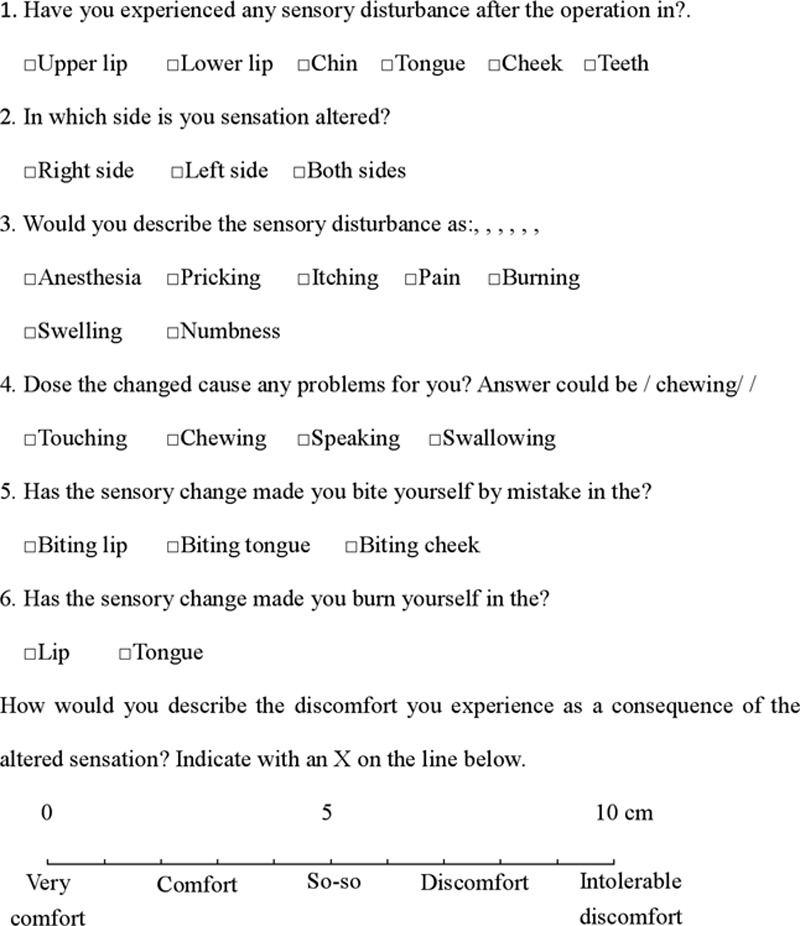
A multiple-choice questionnaire, modified from Al-Bishri et al.,^19^ was administered to patients at each designated time, asking them about any sensory change that they might have noticed along the maxillofacial area. A VAS graded from 0 (no discomfort) to 10 (intolerable discomfort) was included to allow the patients to describe the effect of these changes on their lives.

The questionnaire, modified from a previous study,^19^ comprised 6 questions according to the symptoms and functions of altered sensation. Patients had multiple options for each question, except for the second question, which was a single-choice question (Fig. 1). There were a total of 25 options for the 6 questions. A point was given for each option chosen. The total score of the questionnaire (TSQ) was the sum of chosen options for the 6 questions. A lower TSQ was considered to indicate a better recovery from NSD.

#### VAS

We used the VAS to enable the patients to describe the discomfort they experienced because of the altered sensation. The VAS was a 10-cm horizontal line that was anchored by word descriptors at each end. The end of the left side (0 cm) was defined as (high comfort), the middle of the line was defined as (tolerable discomfort; 5 cm), and the end of the right side (10 cm) was defined as (intolerable discomfort). The patients marked the line at the point they felt represented their perception of their current state. The score was determined by measuring (in millimeters) the distance from the left-hand end of the line to the marked point, with a shorter distance representing a better recovery from NSD.

### Statistical Analysis

We performed statistical analyses by using the statistical software package SPSS version 19.0 for Windows (SPSS Inc., Chicago, Ill.). The patients were due for data collection visits at T0, T1, T2, T3, T4, T5, T6, T7, and T8. We compared sensory recovery using the TSQ and VAS between the control and study groups with a 2-sample *t* test at each time point (T1–T8). We compared the scores from the 3 age groups by using analysis of variance (ANOVA). Bivariate statistics were computed to assess the relationships between study variables. Statistical significance was determined with a *P* value of < 0.05.

## RESULTS

### Patient Characteristics

In total, 123 consecutive patients provided consent. Three patients declined to participate, and 5 were lost to follow-up. We recruited 115 patients (46 men and 69 women) into our study. Their mean age was 23.26 ± 5.19 years, ranging from 18 to 42 years. All patients demonstrated skeletal class III deformities and had undergone bimaxillary OGS (Le Fort I osteotomy and BSSO with or without a genioplasty) performed by a team of 3 attending surgeons.

Table 2 presents the bivariate association between the study variables in the control and study groups. After randomization into 2 groups, the control group comprised 56 patients (32 women and 24 men) with a mean age of 23.2 ± 5.3 years, whereas the study group comprised 59 patients (37 women and 22 men) with a mean age of 24.4 ± 4.6 years. The majority of patients (n = 88, 76.5%) received genioplasty. No statistically significant difference in the measured variables was observed between the 2 groups.

**Table 2.**
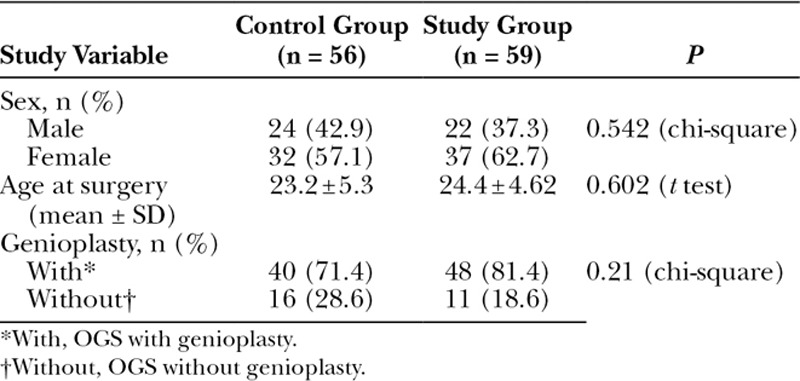
Bivariate Analysis of All Study Variables in the Control and Study Groups

Table 3 presents the bivariate association between the study variables and the primary outcome variables (ie, the TSQ and VAS). All the variables of age, sex, and genioplasty were not significantly associated with the TSQ or VAS.

**Table 3.**
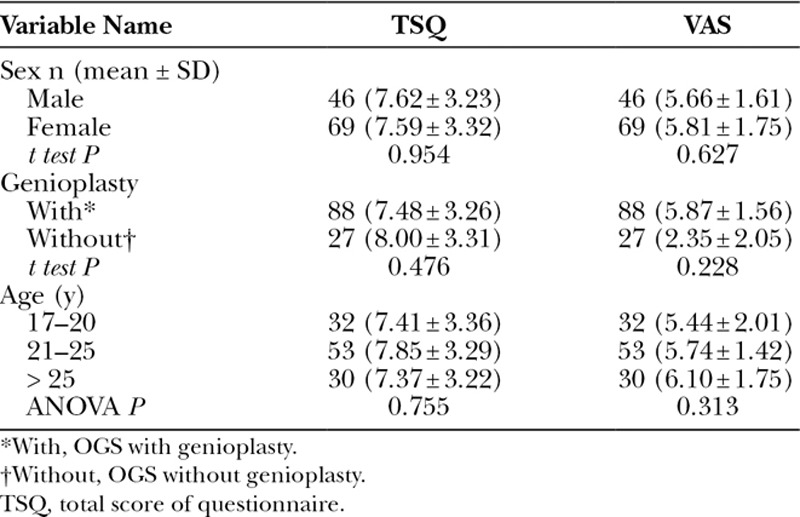
Bivariate Analysis of All Study Variables Versus Primary Outcome Variables (TSQ, VAS)

Table 4 presents the association between the primary predictor (sensory retraining or not) and the primary outcome variables (ie, the TSQ and VAS). Table 4 indicates that the TSQ continually decreased over time after surgery in both groups. Compared with the control group, the study group achieved a lower, but not significantly lower, TSQ at every time point from T3 to T8. The VAS also continuously decreased after surgery (ie, from 5.72 to 2.50 cm in the control group and from 5.78 to 2.49 cm in the study group). However, the VAS was not significantly different between the 2 groups from T0 to T8.

**Table 4.**
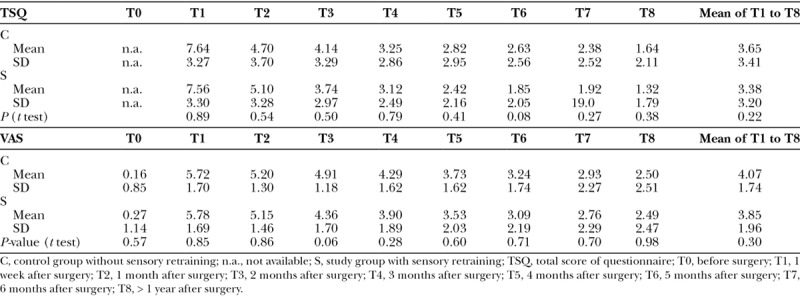
Bivariate Association between Sensory Retraining and Primary Outcome Variables Assessed by TSQ and VAS

**Fig. 2.**
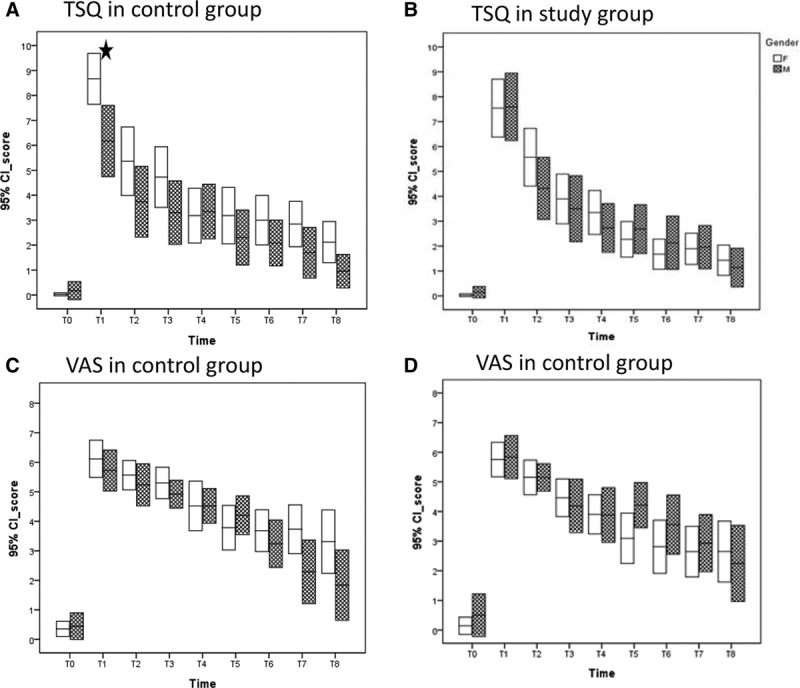
Sex difference represented by mean and 95% confidence interval for male and female patients at each time point from T0 to T8. A, Distribution of TSQ in the control group; B, Distribution of TSQ in the study group; C, Distribution of VAS in the control group; D, Distribution of VAS in the study group, statistically different (*t* test, *P* < 0.05). TSQ, total score of questionnaire.

**Fig. 3.**
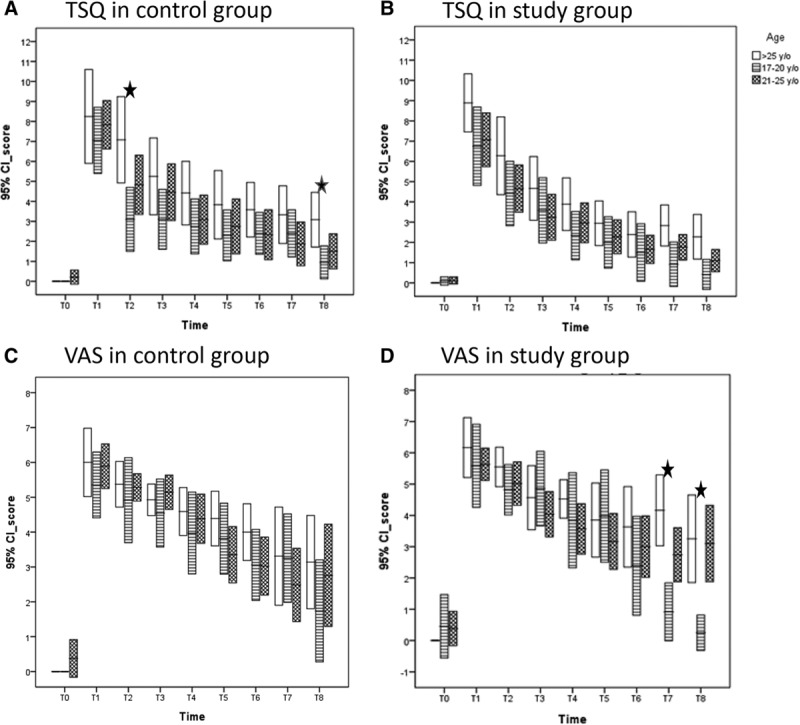
Age difference represented by mean and 95% confidence interval for 3 age groups (> 25, 21–25, and 17–20 years) from T0 to T8. A, Distribution of TSQ in the control group; B, Distribution of TSQ in the study group; C, Distribution of VAS in the control group; D, Distribution of VAS in the study group, statistically different (ANOVA, *P* < 0.05). TSQ, total score of questionnaire.

Table 5 summarizes the results from a linear regression analysis of the bivariate association between all study variables and primary outcome variables (ie, the TSQ and VAS). Sex, age, and genioplasty were significantly (*P* < 0.05) or near significantly (*P* < 0.15) associated with the TSQ and age with the VAS. These 3 variables were isolated separately to identify each effect on NSD. Regarding sex, female patients mostly achieved a higher, but not significantly higher TSQ (Fig. 2A) and VAS (Fig. 2C) than those of their male counterparts in the control group (Fig. 2A, C) after T2. Therefore, women were more sensitive, but not significantly sensitive, to NSD, as compared with men in the control group. Women also responded well to sensory retraining and had lower, but not significantly lower, TSQ (Fig. 2B) and VAS (Fig. 2D) values than their male counterparts from T5 to T7. Regarding age, the oldest patients mostly attained a higher TSQ and VAS than the youngest patients from T1 to T8 in the control group (Fig. 3A, 3C). The oldest patients did not respond well to sensory retraining, and their VAS was significantly higher than that of the youngest patients at T7 and T8 (Fig. 3B, 3D). Regarding genioplasty, sensory retraining made no difference, regardless of whether the patients received genioplasty or not, from T1 to T8.

**Table 5.**
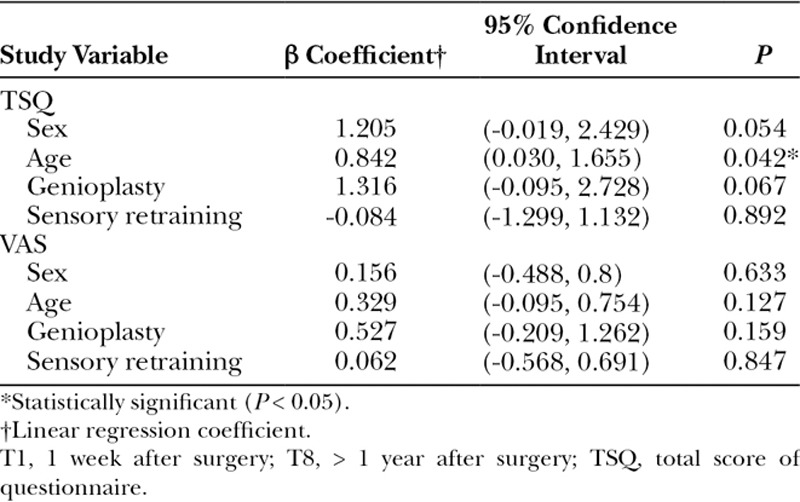
Bivariate Associations between Predictors and TSQ from T1 to T8 and VAS from T1 to T8

## DISCUSSION

In this study, we asked the question whether sensory retraining could facilitate recovery from NSD caused by a BSSO. The observed NSD, assessed by the TSQ and VAS, decreased continually from T1 to T8 in both groups. Sensory retraining facilitated recovery from NSD, as shown by the lower TSQ and VAS in the study group from T3 to T8, but sensory retraining did not significantly improve recovery from NSD throughout the observation. Regarding sex, women were more sensitive to NSD and mostly obtained higher, but not significantly higher, TSQ and VAS values than men after surgery in the control group. Women also responded better to sensory retraining and obtained lower, but not significantly lower, TSQ and VAS values from T5 to T7 in the study group. Concerning age, the oldest patients mostly obtained higher TSQ and VAS values than the youngest patients in the control group. The oldest patients did not respond well to sensory retraining, and their VAS became significantly higher than that in the youngest patients at T7 and T8.

Phillips et al.^20^ conducted a randomized clinical study and instructed the patients with only an open-mouth exercise and a sensory retraining exercise. The sensory retraining exercise included the discrimination of nonmoving touch, the orientation of moving touch, and the direction of moving touch, in that order. In our retraining program, the retraining exercise also involved 3 stages, and the details of the instructions were based on a clinical study regarding the impact of sensory reeducation in patients with hand nerve injury.^21^ The postoperative transient altered sensation of cutaneous distributions is always caused by soft-tissue edema and inflammation or direct or indirect injury to the nerves. The 3 stages of our exercise can relieve facial swelling and offer greater sensory stimulation for sensory recovery. Furthermore, this sensory reeducation can improve a patient’s ability to interpret the altered sensory response from injured nerves, in addition to improving his or her perception of the function. The first stage involved a massage on the affected facial area 1 week to 1 month after surgery, which could help reduce discomfort and the feelings associated with altered sensations caused by facial swelling. The second stage was similar to that in the study of Phillips et al.^11^ and involved training the orientation of moving touch; however, in the final stage, we instructed the patients to discriminate between different objects, which was a modified stage from the sensory rehabilitation of a hand nerve injury.

Our study used a patient-centered survey assessed by the TSQ and VAS to evaluate sensory recovery. Our VAS results show that the degree of discomfort was 5.72–5.78 cm at 1 week after surgery; this figure progressively decreased to 2.76–2.93 cm at 6 months after surgery and to 2.49–2.50 cm at 1 year after surgery. At 6 months after surgery, the patients of both groups felt more comfortable than immediately after surgery; therefore, they indicated a mean VAS score of 2.76–2.93 cm. Travess et al.^23^ also used the VAS to evaluate patient discomfort resulting from postoperative altered sensations. They noted that distress with facial sensory impairment was at 55.3 mm at 1 week after surgery, 38.6 mm at 6 weeks after surgery, and decreased to 30.0 mm at the 26-week follow-up. Ow and Cheung^24^ found that VAS scores were elevated early after surgery, and the scores gradually decreased over time from 6 weeks to 12 months after surgery but did not revert to normal preoperative values. Kim et al.^22^ applied a 10-cm VAS, and they ranked an altered sensation from 10 (completely paralyzed and unable to conduct routine daily life) to 0 (normal). Their result was 5.40 cm at 1 month after surgery, 4.00 cm at 3 months after surgery, and 3.36 cm at 6 months after surgery. They concluded that the VAS of the sensory dysfunction decreased significantly with time.

In this study, the TSQ summarized the overall subjective perception of NSD from various aspects, namely anatomical location, quality of sensory disturbance, and affected oral function. The TSQ can effectively quantify the subjective perception of NSD. Similar to the VAS, the TSQ clearly indicated that NSD progressively decreased with time, and sensory retraining could lessen the subjective impression toward NSD. In contrast to the VAS that documents the single aspect of discomfort, the TSQ could sum up different aspects of subjective perceptions of NSD and offer more robust and stable results to quantify patients’ NSD at each time point throughout the observation.

Although the TSQ and VAS values of female patients were mostly higher than those of their male counterparts from T1 to T8 in the control group, no significant difference emerged between female and male patients after T2. Previous studies have also noted similar results. Nesari et al.^8^ recruited 68 patients who had undergone a BSSO, and they used a questionnaire and sharp-blunt discrimination to evaluate patients at 2, 6, 18, and 30 months after surgery. They found that patient sex did not influence the sensitivity score. Mensink et al.^25^ enrolled 158 patients who had undergone a BSSO, and they used a subjective approach by asking about the feelings of the lower lip; they employed the light touch detection method and pinprick discrimination to conduct the evaluation at 1, 6, and 12 months after surgery. They noted that sex had no influence on hypoesthesia (*P* = 0.985). Chen et al.^26^ examined 274 patients who had undergone a sagittal split osteotomy, and they used a subjective assessment on a 5-point scale and sharp-blunt discrimination to evaluate patients. The results showed no sex-related differences in nerve dysfunction after the sagittal split osteotomy. However, other studies have yielded conflicting results. Yoshioka et al.^27,28^ have recruited 28 and 35 patients with mandibular prognathism, and they used computed tomography images to assess the distance from the buccal aspect of the IAN canal to the outer buccal cortical margin of the mandible. They found that women were significantly more likely than men to experience NSD of the IAN after a sagittal splitting ramus osteotomy.

Regarding age, the oldest patients in this study obtained higher TSQ and VAS values than the youngest patients in both groups from T1 to T8. This result is consistent with those reported in previous studies, which have identified age as a risk factor. Ylikontiola et al.^29^ recruited 30 patients who had undergone a BSSO, and they conducted a follow-up 1 year after surgery. They used a self-administered questionnaire to evaluate the patients and found that patients aged ≤ 30 years had fewer neurosensory problems than patients aged 30 years or older during the follow-up (*P* = 0.039). Espeland et al.^30^ recruited 516 patients who underwent surgery, and they used a questionnaire to evaluate sensory impairment at a 3-year follow-up. They noted that the age at the time of surgery was significantly associated with a disturbed sensory function (*P* = 0.001). Phillips et al.^31^ examined 186 patients who underwent a BSSO, and they conducted a follow-up 2 years after surgery. They performed a subjective assessment using a questionnaire and found that age was significantly associated with the patient reporting altered sensations after surgery. The results of these previous studies support the finding that older patients are more likely to report functional deficits associated with altered sensations.

## CONCLUSIONS

NSD, assessed by the TSQ and VAS, continually decreased over time after a BSSO, regardless of whether patients received sensory retraining or not. The study group experienced less discomfort from NSD than the control group from T1 to T8, but no significant difference emerged between the 2 groups. The sex difference for sensory retraining was not statistically different throughout the observation period. The oldest patients consistently attained higher TSQ and VAS values than the youngest patients from T1 to T8 and did not respond well to sensory retraining, particularly at T7 and T8.

## ACKNOWLEDGMENTS

The authors thank Pei Ju Lin for her invaluable help for statistical analysis and Amy Cheng for her assistance in obtaining patient records for this study. This study was supported by Chang Gung Memorial Hospital (CRRPG5C0223, CRRPG5C0263) and the Ministry of Science and Technology, Taiwan (101-2314-B-182-050). This article was edited by Wallace Academic Editing.
